# Surface Structure and Anion Effects on Electrooxidation of Isopropanol on Pt(*hkl*)

**DOI:** 10.1021/acs.jpclett.5c02114

**Published:** 2025-08-23

**Authors:** Ao Li, Gabriel Melle, Camilo A. Angelucci, Enrique Herrero, Changwei Pan, Vinicius Del Colle, Qingyu Gao

**Affiliations:** † College of Chemical Engineering, 12392 China University of Mining and Technology, Xuzhou 221116, People’s Republic of China; ‡ Instituto de Electroquímica, 16718 Universidad de Alicante, Apdo. 99, E-03080 Alicante, Spain; § 74362 Federal University of ABC, Center for Natural and Human Sciences, Av. dos Estados, 5001, 09210-580 Santo André, São Paulo, Brazil; ∥ Aeronautics Technological Institute, Chemistry Department, Praça Marechal Eduardo Gomes, 50 Vila das Acácias, 12228-900 São José dos Campos, São Paulo, Brazil

## Abstract

This study focuses on the electro-oxidation of isopropanol on low-index platinum single-crystal surfaces—Pt(111), Pt(110), and Pt(100)—in acidic electrolytes containing either sulfuric acid (H_2_SO_4_) or perchloric acid (HClO_4_). The aim is to elucidate the roles of crystallographic orientation and electrolyte anions in the reaction pathway and associated dynamic instabilities. While conventional voltammetric and spectroscopic techniques provide insights into reaction products and adsorbed intermediates, galvanostatic experiments are employed here to probe the emergence of potential oscillations, which serve as sensitive indicators of nonsteady-state surface processes. The results reveal a marked dependence of oscillatory behavior on both the electrode surface structure and the electrolyte composition. Pt(111) exhibits no oscillations under any of the tested conditions, consistent with a direct oxidation pathway that predominantly yields acetone and results in negligible accumulation of strongly adsorbed intermediates. Pt(110) displays limited and transient oscillations only in perchloric acid, suggesting a minor role for adsorbed poisoning species under these conditions. In contrast, Pt(100) shows robust and sustained potential oscillations across a wide range of current densities in both electrolytes, indicating a mechanistic regime dominated by the indirect pathway involving the formation and oxidation of adsorbed CO (CO_ads_). Moreover, the low sensitivity of oscillations on Pt(100) to the nature of the electrolyte anion suggests that the dynamics of CO_ads_ buildup and removal are primarily dictated by the surface atomic arrangement rather than by competitive anion adsorption. These findings also underscore the utility of galvanostatic potential oscillations as a powerful diagnostic tool for detecting adsorbed intermediates that may remain elusive under steady-state conditions.

## Graphical abstract

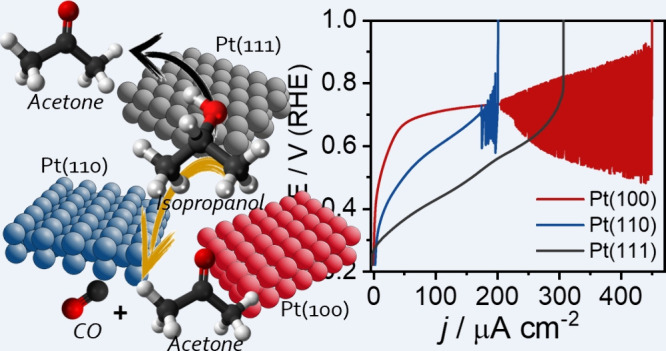

The electro-oxidation of small organic molecules with a three-carbon backbone, such as C3 alcohols, has attracted significant attention due to their high theoretical energy densities, low toxicity, and ease of handling, as they are typically liquid at ambient conditions.^1^ Within this class, C3 alcohols like 1-propanol, isopropanol (2-propanol), and polyols (e.g., glycerol, 1,2-propanediol, and 1,3-propanediol) serve as valuable molecular probes for investigating the electrocatalytic behavior of different metallic surfaces.^2,3^ These molecules allow for the systematic investigation of how subtle differences in their structure influence adsorption modes, surface poisoning, intermediate species formation, and overall reactivity.^3–6^

Among these compounds, isopropanol holds particular relevance as the simplest secondary alcohol, offering a structurally minimal yet mechanistically rich model for understanding the influence of the effect of the adsorbed species present on the surface on electrochemical reactivity. As previously observed for simpler alcohols such as methanol^7,8^ and ethanol,^9^ adsorbed hydroxyl species (OH_ads_) play a critical role in facilitating both the adsorption and subsequent oxidation of isopropanol.^10^ On platinum electrodes, the primary oxidation product is acetone, formed via a two-electron dehydrogenation pathway, with CO_2_ generation observed only at higher potentials where surface oxides are involved.^11–14^

The role of crystallographic orientation in modulating the oxidation of isopropanol in sulfuric acid solution was first systematically addressed by Sun et al. in the 1990s through in situ time-resolved FTIR spectroscopy.^13^ The results demonstrated that while the overall mechanism remains consistent across low-index and stepped surfaces, the reaction kinetics and product distribution exhibit structure sensitivity. Acetone and CO_2_ were the dominant products, with no evidence of CO formation, indicating the absence of C–C bond cleavage. These findings were corroborated by subsequent FTIR and DEMS studies on polycrystalline Pt, reinforcing the idea of a nondissociative oxidation pathway under typical conditions.^15^

However, this paradigm was recently challenged by Mekazni et al.,^14^ who, using Pt(100) electrodes, detected CO formation, implying the occurrence of C–C bond scission, a pathway previously considered improbable for isopropanol oxidation. This breakthrough revived discussions on the structure–activity selectivity relationship, emphasizing that the oxidation pathway is more nuanced than previously assumed and is heavily modulated by surface ensemble effects and electrolyte composition.

In parallel, advanced methodologies in nonlinear dynamics, particularly the analysis of kinetic instabilities such as potential oscillations, have emerged as valuable tools for probing mechanistic intricacies in electrooxidation reactions.^16–21^ Unlike steady-state measurements, oscillatory behaviors are highly sensitive to intermediate accumulation, surface coverage, and feedback mechanisms, making them excellent indicators of hidden reaction steps. For example, Ragassi et al.^16^ demonstrated that in the galvanostatic oxidation of isopropanol in the presence of methanol, self-organized potential oscillations arise due to competitive adsorption of isopropanol and acetone, despite the absence of CO as a major poisoning species. Notably, under certain conditions, such oscillations may even enhance catalytic performance.

Despite the growing body of research on the electrochemical oxidation of isopropanol, studies explicitly focusing on kinetic instabilities on well-defined Pt­(*hkl*) surfaces, especially under galvanostatic conditions, remain scarce. Moreover, the role of electrolyte composition, specifically sulfuric versus perchloric acid, which differ markedly in their anion adsorption behavior, on oscillatory patterns and mechanistic pathways has yet to be fully elucidated. Therefore, the present study aims to systematically explore the emergence of potential oscillations during the galvanostatic electro-oxidation of isopropanol on Pt single crystals in sulfuric and perchloric acid media. By mapping the oscillatory regimes across crystallographic orientations, we will highlight the surface- and electrolyte-dependent reaction pathways and provide additional understanding of intermediate dynamics and poisoning mechanisms. This approach serves as a complementary mechanistic probe to traditional spectroscopic techniques and has the potential to reveal hidden complexities in electrocatalytic alcohol oxidation.

## Experimental Section

### Materials and Methods

Single-crystal platinum electrodes with low-index orientations, Pt­(*hkl*), were prepared from spherical platinum beads (∼2.0 mm in diameter) according to the protocol established by Clavilier.^22^ Before each experiment, the electrodes were cleaned by flame annealing in a mixed oxygen–hydrogen flame, followed by cooling under a H_2_/Ar atmosphere. The surface orientation and cleanliness of the Pt electrodes were verified by cyclic voltammetry (CV) in a base electrolyte containing 0.1 mol L^–1^ H_2_SO_4_ or HClO_4_.^23,24^ Characteristic features of the hydrogen adsorption/desorption region and the voltammetric profiles between 0.05 and 0.80–0.90 V were used as diagnostic indicators for electrode quality and solution cleanliness.

All experiments were conducted in a conventional three-electrode glass cell at 25 °C. A platinum foil (geometric area: 1.0 cm^2^) served as the counter electrode, and a reversible hydrogen electrode (RHE) was used as the reference. All potentials reported in this work are referenced against the RHE scale. Electrochemical measurements were performed using a Metrohm Autolab PGSTAT302N potentiostat.

CVs were conducted in the potential range of 0.05 to 0.80–0.90 V at a scan rate of 50 mV s^–1^. Typically, four consecutive scans were recorded to assess the stability and evolution of the electrooxidation process. Following the voltammetric characterization, galvanodynamic and galvanostatic experiments were performed to probe kinetic instabilities and oscillatory behavior under current-controlled conditions. Galvanodynamic scans were performed by linearly increasing the current, while galvanostatic chronoamperometry was carried out at fixed current densities.

All reagents used were of analytical grade: sulfuric acid and perchloric acid (Sigma-Aldrich, ≥99.999% trace metal basis), isopropanol (Sigma-Aldrich, ≥99.9%), and ultrapure water (18.2 MΩ cm) from a Millipore Milli-Q system.

Figure 1 presents voltammetric profiles recorded on Pt(111), Pt(110), and Pt(100) single-crystal electrodes in electrolytes containing 0.2 mol L^–1^ isopropanol and 0.1 mol L^–1^ HClO_4_ or H_2_SO_4_. The voltammetric profiles demonstrate that isopropanol electrooxidation is highly dependent on both the crystallographic orientation of the platinum surface and the nature of the electrolyte. In perchloric acid solutions, the Pt(111) and Pt(110) electrodes exhibit significantly higher current densities than in sulfuric acid. On Pt(111), the current–potential curve shows the typical shape of an irreversible diffusion-controlled process: after the peak potential, the current decays proportionally to (t – t_0_)^1/2^, consistent with diffusion-limited transport of isopropanol to the electrode surface.^14^ Despite similar onset potentials (∼0.28 V) in both media, the peak currents in sulfuric acid are approximately 12 times lower, primarily due to the strong adsorption of sulfate anions, which inhibit the formation of OH_ads_. Since OH_ads_ plays a key catalytic role in isopropanol oxidation, its suppression by sulfate adsorption significantly reduces the overall reactivity. In contrast, perchlorate anions exhibit negligible specific adsorption, allowing the formation of OH_ads_ and leaving Pt active sites accessible for isopropanol oxidation.^23,24^

**Figure 1:**
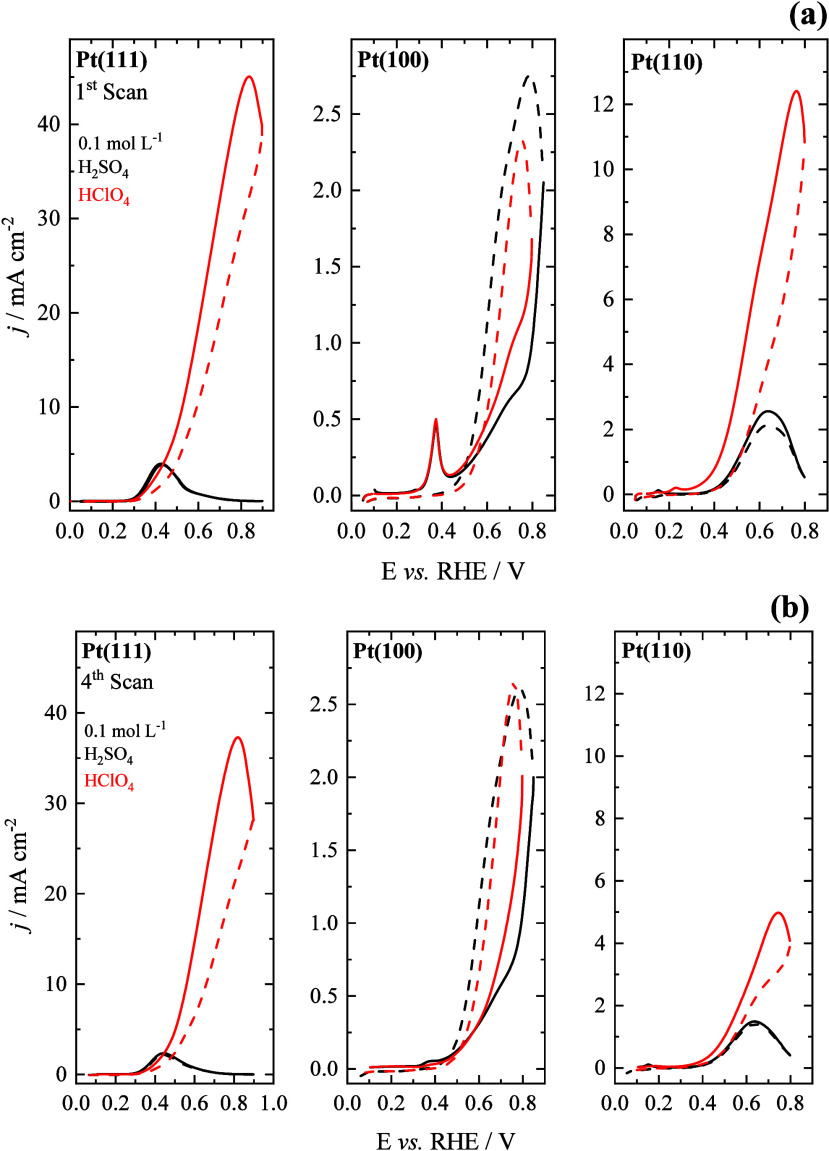
Voltammetric profiles for 0.2 mol L^–1^ isopropanol electrooxidation in acid media (0.1 mol L^–1^ H_2_SO_4_/HClO_4_) for the Pt(111), Pt(100), and Pt(110) electrodes: (a) 1st and (b) 4th scan. The full and dashed lines represent the positive and negative scan directions, respectively. Scan rate: 0.05 V s^–1^.

On Pt(100), the influence of the electrolyte is markedly less pronounced. In both perchloric and sulfuric acids, the voltammetric profiles are similar and exhibit lower current densities than those observed on Pt(111) and Pt(110). A distinct anodic peak around 0.38 V is observed, which is attributed to the formation of adsorbed CO, a result of early C–C bond cleavage.^14^ This behavior is atypical for the oxidation of small organic molecules, where strong adsorption of sulfate anions generally suppresses reactivity by blocking active sites.^25–29^ Indeed, the inhibitory effect of sulfate is manifested on Pt(111) and Pt(110), consistent with prior studies identifying sulfate as a strong surface-blocking agent.^28–30^ In contrast, the nonspecifically adsorbed perchlorate anions and the availability of OH_ads_ enable significantly higher reactivity, especially on Pt(111). This remains true even at potentials above the reported potential of zero total charge (pztc) for Pt(100) in acidic media,^24,31^ suggesting that CO formation is the rate-controlling pathway on the Pt(100) surface under these conditions.

Beyond the competition between anions for surface sites, it is important to highlight the distinct reaction mechanism observed on Pt(100), characterized by its ability to selectively cleave the C–C bond and form adsorbed CO as a stable intermediate. Remarkably, this pathway appears largely insensitive to variations in the supporting electrolyte. This observation aligns with recent literature identifying Pt(100) as particularly active toward dissociative adsorption and C–C bond cleavage reactions,^14^ although prior studies have not systematically explored the role of anion competition during isopropanol oxidation. The presence of CO at low potentials, clearly detected around 0.38 V by in situ FTIR, strongly supports this mechanistic pathway.

Further insight into the formation and accumulation of strongly adsorbed intermediates, such as CO or other strong adsorbates, can be obtained by analyzing hysteresis between the positive and negative scan directions in cyclic voltammetry. For Pt(111) and Pt(110), the current densities in the reverse scan are lower than those in the forward scan, primarily due to isopropanol depletion in the diffusion layer. Moreover, the voltammetric profiles in the hydrogen adsorption/desorption region are nearly identical to those obtained in the absence of isopropanol, indicating that no significant poisoning occurs. This observation is consistent with previous FTIR and DEMS studies, which showed that acetone is the dominant product on Pt(111) and Pt(110), with negligible formation of poisoning species.^14,32^ In contrast, Pt(100) exhibits pronounced hysteresis in both electrolytes, with currents in the positive scan direction significantly lower than those recorded in the negative one, suggesting substantial accumulation of strongly adsorbed intermediates, primarily CO.

To further investigate these surface-dependent mechanistic features, galvanodynamic techniques were employed to explore dynamic instabilities not accessible through conventional steady-state electrochemical methods. The application of a time-dependent current enables the study of transient states and nonstationary regimes, providing valuable insight into intermediate formation, reaction feedback, and surface poisoning phenomena. Figure 2 presents galvanodynamic curves obtained by scanning the current density at 0.20 μA s^–1^ cm^–2^ in 0.10 mol L^–1^ HClO_4_, highlighting the potential regions where oscillatory behavior emerges. Equivalent measurements performed in 0.10 mol L^–1^ H_2_SO_2_ are presented in Figure S1.

**Figure 2:**
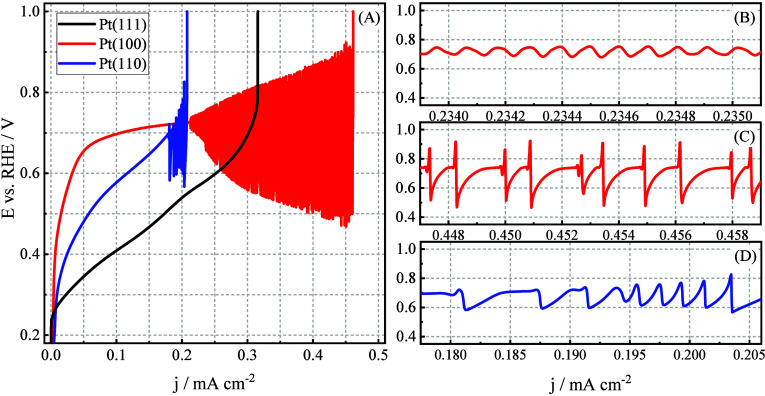
(A) Galvanodynamic curves, obtained at 0.20 μA s^–1^ cm^–2^, for the isopropanol oxidation on the Pt(111), Pt(100), and Pt(110) electrodes, in 0.10 mol L^–1^ HClO_4_. (B–D) Potential instabilities observed on the (B and C) Pt(100) and (D) Pt(110) electrodes.

The results presented in Figure 2 and Figure S1 reveal distinct behaviors in the electrooxidation of isopropanol, influenced by both the crystallographic orientation of the Pt surface and the type of supporting electrolyte. On Pt(111), a monotonic increase in potential with increasing current density is observed in both perchloric and sulfuric acid, without any indication of potential oscillations. In contrast, both Pt(100) and Pt(110) exhibit galvanodynamic instabilities, with Pt(100) displaying a broad oscillatory region between approximately 0.45 and 0.95 V in both electrolytes. This behavior suggests that, for Pt(100), the anion of the supporting electrolyte does not significantly affect the occurrence of oscillations. In the case of Pt(110), however, the supporting electrolyte exerts a notable influence on the oscillatory dynamics. In perchloric acid, oscillations are observed over a narrower current range compared to Pt(100), spanning approximately 0.59 to 0.81 V after a brief induction period. In sulfuric acid, by contrast, only a single oscillatory cycle is detected around 0.7 V across the entire curve (Figure S1), indicating a strong suppression of oscillatory behavior.

A further point of interest lies in the differences between the oscillation morphologies observed on Pt(100) and Pt(110) in perchloric acid, more clearly depicted in panels B–D of Figure 2 and Figure S1. On Pt(100), oscillations initially emerge with period-one (P1) behavior in a nearly harmonic fashion, and subsequently evolve into more complex mixed-mode oscillations with less regular morphology. In contrast, oscillations on Pt(110) appear initially as mixed-mode and later transition into period-one oscillations with a nonharmonic profile. These are shorter in duration and confined to a narrower potential range compared to those on Pt(100). While this is the first report of oscillatory dynamics during isopropanol oxidation on Pt single-crystal surfaces, the galvanodynamic profiles for Pt(111) and Pt(110) resemble those previously observed by Del Colle et al.^30^ during glycerol oxidation under oscillatory regimes.

As discussed earlier, the emergence of oscillations in organic molecule oxidation is typically attributed to the dynamic competition between adsorbed reactive and inactive species within the same potential range.^33,34^ This suggests that the oscillatory behavior observed on Pt(100) and Pt(110) results from the formation of distinct surface species not present on Pt(111). In the specific case of isopropanol oxidation, the present results, supported by FTIR analyses,^14^ indicate that the oscillations on Pt(100) and Pt(110) are associated with C–C bond cleavage and the consequent formation of adsorbed CO. Conversely, on Pt(111), the oxidation proceeds predominantly via acetone formation without CO adsorption, explaining the absence of dynamic instabilities. It should also be emphasized that acetone, the main oxidation product of isopropanol, strongly adsorbs on both Pt(100) and Pt(110) surfaces.^35,36^ However, on Pt(110), adsorbed acetone is not oxidized below 0.9 V and therefore cannot contribute to the oscillatory regime. In contrast, on Pt(100), acetone not only adsorbs but also dissociates to form CO_ads_, which is readily oxidized around 0.8 V. This behavior establishes the feedback conditions necessary for the emergence of oscillations on the Pt(100) surface.

To further investigate the role of surface structure in oscillation emergence during isopropanol oxidation, galvanostatic time series (GTS) experiments were conducted. These measurements, performed at constant current, provide insight into oscillation periodicity and dynamic evolution. The applied current densities were selected based on a normalization approach used in previous studies^21,27,37,38^ involving other organic molecules and electrode surfaces. In this normalization procedure, the applied current is the central current between the initial and final currents of the potential oscillations of the curves obtained during the linear current sweep (galvanodynamic curve showed in Figure 2), that is, the central current between the first and last points where the current sweep leads to a decrease in the potential.

Although Pt(111) was tested under several current densities, no oscillatory behavior was detected. Similarly, Pt(110) exhibited no oscillations in sulfuric acid, while only irregular, nonperiodic oscillations were observed in perchloric acid (Figure S2), consistent with the limited hysteresis seen in the corresponding cyclic voltammograms. In contrast, Pt(100) displayed well-defined oscillatory behavior in both electrolytes (Figures S3A–B), corroborating the galvanodynamic results shown in Figure 2 (panels A–C).

To characterize the periodicity of the oscillations observed on Pt(100), return maps^39,40^ were constructed from the galvanostatic time series (Figure 3, panels 1a–3b). A return map is a tool from dynamical systems analysis that enables a more concise and informative representation of the temporal evolution of oscillatory signals. In this context, the maps were built using the successive values of the local minima of the potential, allowing for the identification of periodic, quasi-periodic, or chaotic patterns. This approach facilitates the visualization and interpretation of the system’s dynamic structure, providing a clearer description of the underlying oscillatory behavior.

**Figure 3:**
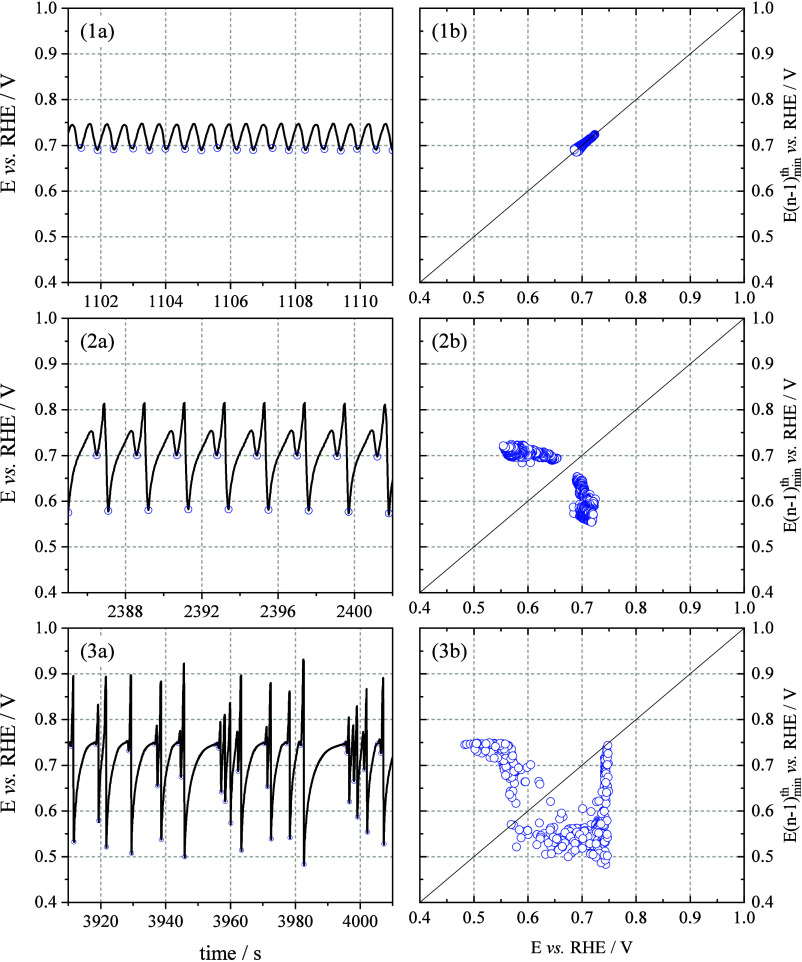
Column (a): Samples of oscillation patterns obtained from the galvanostatic time series at 0.21 mA cm^–2^ for isopropanol electrooxidation reaction on the Pt(100) electrode. Column b: Corresponding return maps for each pattern.

As shown in Figure 3(1a–1b), after an initial induction period, the oscillations on Pt(100) emerge with a nearly harmonic waveform and a periodicity of one, centered around 0.72 V with an amplitude of approximately 50 mV. These then evolve into period-two oscillations (Figure 3(2a)), which display a larger amplitude of about 0.23 V and occur within a potential range of 0.57 to 0.80 V. These are characterized by a gradual increase in potential followed by a sharp drop. Over time, oscillations with different periodicities accumulate (Figure 3(3a)), leading to a mixed-period regime with an amplitude of roughly 0.4 V, as reflected in the corresponding return map (Figure 3(3b)). These dynamic instabilities align closely with potential regions previously associated with the formation and oxidation of adsorbed CO, as confirmed by in situ FTIR studies.^14^

The preferential CO formation and adsorption on Pt(100) can be rationalized by its favorable geometry and lower atomic coordination, which contribute to dissociative adsorption pathways and provide higher CO binding energies compared to Pt(111) and Pt(110) surfaces. In agreement with previous in situ FTIR and voltammetric studies, acetone—the primary oxidation product of isopropanol—readily dissociates to COads on Pt(100) but not on Pt(110) below ∼ 0.9 V, whereas Pt(111) follows a nondissociative route that yields acetone without detectable CO formation.^14,35,36^ These differences are intrinsic to the crystallographic orientation and persist across electrolytes, indicating that the enhanced CO_ads_ coverage on Pt(100) is not primarily a result of surface roughness or defects introduced during electrode preparation.

From a nonlinear dynamics perspective, the progression of oscillatory regimes observed on Pt(100) (period-one → period-two → mixed-mode) reflects a slow–fast feedback loop between the coverage of strongly adsorbed intermediates (θ_CO_ and, to a lesser extent, θ_OH/oxide_) and the reaction rate, a mechanism well described in electrochemical oscillator theory.^33,34^ In this framework, the gradual amplitude increase is associated with deeper surface poisoning before each oxidative removal event, leading to larger potential excursions. The broader amplitudes in H_2_SO_4_ compared to HClO_4_ likely stem from reduced steady-state θ_CO_ due to competitive sulfate adsorption, which requires more extensive surface oxidation to trigger CO removal. Oscillations on Pt(100) were robust and reproducible in morphology across repeated galvanostatic runs in both electrolytes, consistent with a structurally governed CO-mediated pathway.^4,14,19,37,43^ The observed spike-like mixed-mode oscillations and history-dependent recovery also mirror behaviors described in neuromorphic systems, where excitable dynamics emerge from similar feedback between slow inhibitory and fast excitatory variables.^6,33,34^ Another interesting point that contributes to amplitude increase is the adsorbate-induced Pt-restructuring. The presence of adsorbates can alter the relative stability of different surface facets, causing a restructuring cycle by cycle and thus exposing some defects that contribute to CO formation.

The limited number of oscillations observed on Pt(110) (Figure S2) and their complete absence on Pt(111), both of which do not show CO formation in short-term voltammetric studies, strongly support the conclusion that the indirect oxidation pathway involving CO_ads_ is primarily responsible for the oscillatory behavior observed on Pt(100).

A similar phenomenon has been reported for glycerol oxidation, both in acidic^4,19,28^ and alkaline^39^ media, where the emergence of oscillations was shown to be limited by mass transport constraints. In these cases, partial oxidation of glycerol feeds the formation of CO on the platinum surface, leading to the conditions necessary for oscillatory behavior.

Under galvanostatic conditions, the oxidation of isopropanol reveals the formation and removal of strongly adsorbed intermediates that are often difficult to detect or distinguish using standard cyclic voltammetry or spectroscopic techniques. These intermediates, typically CO, acetone-derived species, or other partially oxidized organic residues, can periodically poison the catalyst surface, giving rise to characteristic oscillatory potential responses. As such, galvanostatic experiments serve as sensitive probes for the dynamic accumulation and subsequent oxidation of these strongly adsorbed species, with oscillations in electrode potential providing indirect evidence of their presence.

Notably, CO_ads_ detection reported by Mekazni et al.^14^ in perchloric acid contrasts with the earlier findings by Sun and Lin,^13^ who reported no CO_ads_ formation during isopropanol oxidation in sulfuric acid. While this might initially suggest that sulfate adsorption significantly alters the reaction pathway, the oscillatory behavior observed on Pt(100) in both electrolytes in the present study indicates that CO formation is an intrinsic feature of the Pt(100) surface, rather than being dictated by the nature of the electrolyte anion. A critical factor that may explain this discrepancy lies in the experimental protocol used by Sun and Lin,^13^ in which the Pt­(*hkl*) electrodes were cooled in air before measurements. This procedure likely induced surface oxidation and restructuring, generating defects and heterogeneities.^41^ Such surface irregularities could alter the electrocatalytic behavior, potentially promoting direct oxidation pathways or hindering the formation of CO_ads_ species. This may account for the absence of a detectable infrared band for CO_ads_ in their in situ FTIR measurements.

The galvanostatic results presented here underscore the importance of surface structure in determining the oscillatory dynamics of isopropanol oxidation. For Pt(100), the indirect oxidation pathway involving CO_ads_ drives the emergence of potential oscillations, largely independent of the specific anion present in the supporting electrolyte. This behavior highlights a clear structure–activity relationship, with Pt(100) exhibiting a distinct mechanism compared to Pt(111) and Pt(110).

This interpretation contrasts with the conclusions of Ragassi et al.,^42^ who attributed oscillations during isopropanol oxidation to the accumulation and removal of isopropanol-derived intermediates and acetone, rather than CO_ads_. They reported that CO was less prominent than in other small organic molecule oxidations on platinum surfaces. In agreement with their findings, Pt(111) and Pt(110) in the present study predominantly produce acetone and show no oscillatory behavior, consistent with minimal CO formation. However, Pt(100), which facilitates significant CO_ads_ formation, consistently exhibits oscillations, reinforcing the view that CO_ads_ is the critical species driving the dynamic instabilities on this surface.

Consistent with the findings of Nagao et al.,^43^ who investigated the methanol oxidation reaction, the present galvanostatic experiments, when considered alongside in situ FTIR results in perchloric acid,^14^ offer enhanced mechanistic insight into the oscillatory dynamics of isopropanol oxidation on Pt single-crystal electrodes. Nagao et al.^43^ demonstrated that the indirect pathway involving adsorbed CO species displays low sensitivity to the nature of supporting electrolyte anions, as shown in both sulfuric and perchloric acid media. This trend was further corroborated by Melle et al.,^44^ who observed similar behavior in phosphoric acid, reinforcing the electrolyte-independent character of the CO-mediated pathway. These observations align closely with the results reported here.

The potential oscillations recorded in this study reflect the periodic accumulation and oxidative removal of strongly adsorbed intermediates, a dynamic pattern that closely mirrors that described for methanol electrooxidation.^21,27,43,45^ On Pt(100), the oscillatory behavior is predominantly governed by the cyclic oxidation of CO_ads_ to CO_2_ on the electrode surface, occurring in both electrolytes. Notably, the broader oscillation amplitudes observed in sulfuric acid suggest a lower CO_ads_ surface coverage, likely due to the competitive adsorption of sulfate anions. Based on the galvanostatic behavior described above, a mechanistic scheme summarizing the oscillatory electrooxidation pathways of isopropanol on Pt(111), Pt(100), and Pt(110) surfaces is proposed and presented in Scheme 1.

**Scheme 1:**
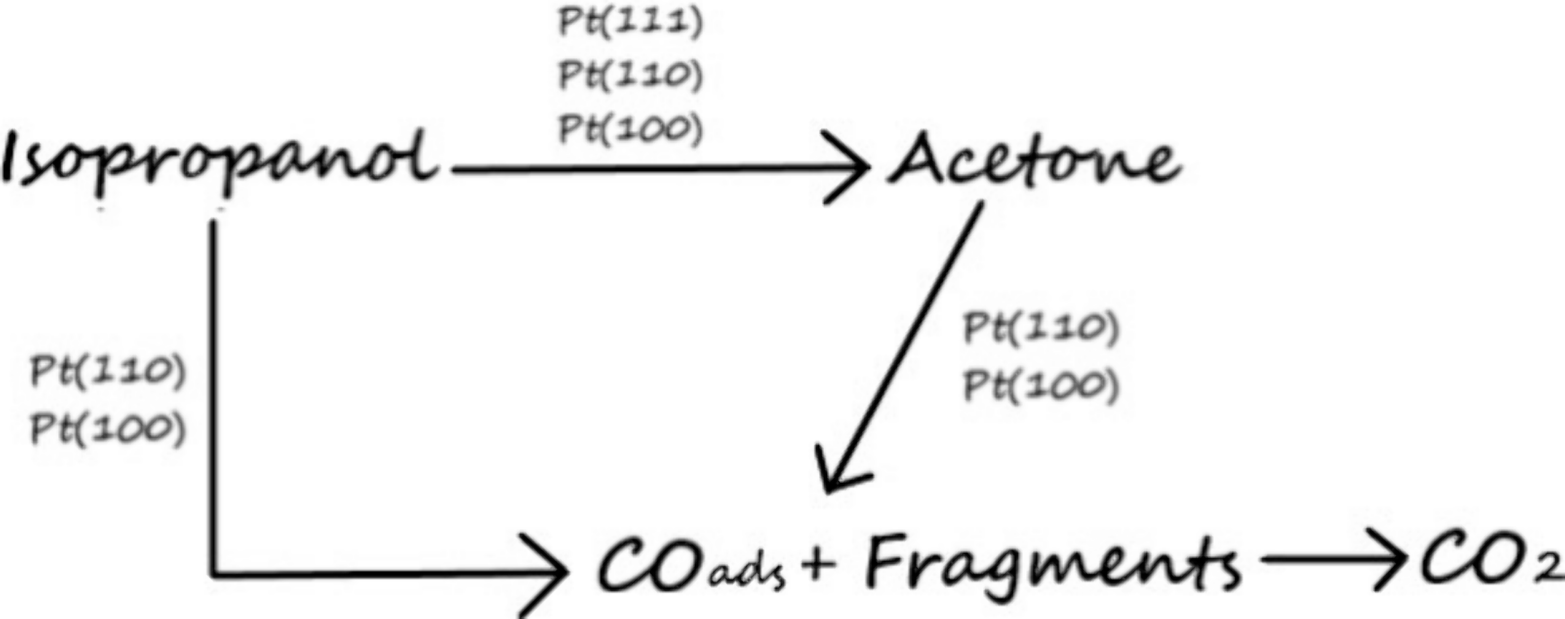
Proposed Reaction Pathways for Oscillatory Isopropanol Electrooxidation on Pt­(*hkl*)

In this study, we conducted a comprehensive comparative analysis of the electro-oxidation of isopropanol on Pt­(*hkl*) in acidic electrolytes with different supporting anions: sulfuric acid (H_2_SO_4_) and perchloric acid (HClO_4_). Our primary objective was to elucidate the combined influence of surface crystallographic orientation and electrolyte anion nature on the reaction mechanisms and dynamic behavior of the system, utilizing the analysis of kinetic instabilities, particularly potential oscillations under galvanostatic conditions, as a sensitive probe for intermediate dynamics and poisoning processes.

Analysis of kinetic instabilities under galvanostatic conditions corroborated and expanded the observations obtained from traditional electrochemical techniques such as cyclic voltammetry and *in situ* FTIR. The oscillatory behavior was shown to be intrinsically linked to the crystallographic orientation. Pt(111) did not exhibit potential oscillations in either electrolyte. Pt(110) displayed a limited oscillatory region with no clear periodicity exclusively in perchloric acid. In stark contrast, Pt(100) demonstrated pronounced and well-defined potential oscillations over a broad range of applied currents, and, crucially, independently of the electrolyte nature (sulfuric or perchloric acid).

The emergence of potential oscillations on Pt(100) in both electrolytes confirms that the indirect pathway involving CO_ads_ formation is structurally governed, rather than being modulated solely by the nature of the electrolyte anion. The absence of significant oscillations on Pt(110) and Pt(111), surfaces that do not promote significant CO_ads_ formation, strongly indicates that the indirect oxidation pathway mediated by CO_ads_ is the primary driver of the observed oscillatory behavior on Pt(100). These findings contrast with some previous interpretations that minimized the involvement of CO in isopropanol.

Overall, the results demonstrate that the oscillatory behavior during isopropanol oxidation is closely linked to crystallographic orientation and intermediate formation pathways. The Pt(100) facet uniquely enables dynamic instabilities through CO_ads_-mediated mechanisms, even in electrolytes containing strongly adsorbing anions like sulfate. These findings provide additional insight into the structure–activity selectivity relationships in C3 alcohol electro-oxidation and are fundamental for understanding kinetic instabilities in complex electrocatalytic systems. The use of potential oscillations as a mechanistic probing tool revealed the importance of the indirect pathway via CO_ads_ for the reaction dynamics on Pt(100), contrasting with the predominantly direct behavior on Pt(111) and Pt(110).

## Supplementary Material



## Notes

Galvanodynamic curves for the isopropanol oxidation on Pt­(*hkl*) and galvanostatic time series of isopropanol electrooxidation reaction on Pt(110) and Pt(100) surfaces (PDF)
