# Supplementary material for: Surface Structure and Anion Effects on Electrooxidation of Isopropanol on Pt(hkl)
Source: J Phys Chem Lett. 2025 Aug 23;16(35):8969–76. doi: 10.1021/acs.jpclett.5c02114 (PMC12415890; doi:10.1021/acs.jpclett.5c02114)
Supplement: Supplementary file 1 [file jz5c02114_si_001.pdf]

Supporting Information

Surface Structure and Anion Effects on Electrooxidation of Isopropanol on Pt(*hkl)*

Ao Li[a], Gabriel Melle[b], Camilo A. Angelucci[c], Enrique Herrero[b], Changwei Pan[a*], Vinicius Del Colle[d*], Qingyu Gao[a*]

[a]*College of Chemical Engineering, China University of Mining and Technology at Xuzhou, 221116, People's Republic of China*

[b]*Instituto de Electroquímica, Universidad de Alicante, Apdo. 99, E-03080 Alicante, Spain*

[c]*Federal University of ABC, Center for Natural and Human Sciences, Av. dos Estados, 5001, 09210-580, Santo André, São Paulo, Brazil*

[d]*Aeronautics Technological Institute, Chemistry Department, Praça Marechal Eduardo Gomes, 50 Vila das Acácias, 12228-900, São José dos Campos, São Paulo, Brazil*

****

Table of Contents:

1. Galvanodynamic curves for the isopropanol oxidation on Pt(hkl) ……………………………2

2. Galvanostatic time series of isopropanol electrooxidation reaction on Pt(110) surface ……………………………………………………………………………………………………3

3. Galvanodynamic time series of isopropanol electrooxidation reaction on Pt(100) surface ……………………………………………………………………………………………………4

1. Galvanodynamic curves for the isopropanol oxidation on Pt(hkl)

Figure S1 presents the studies of galvanodynamic curves for the isopropanol electrooxidation reaction on Pt(111), Pt(100), and Pt(110).

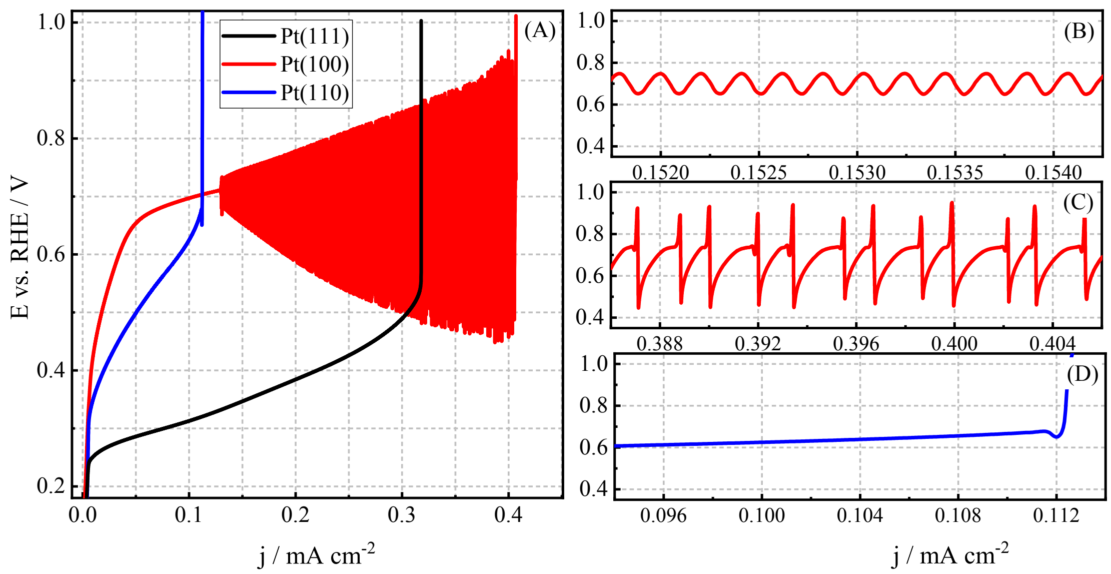

Figure S1. (A) Galvanodynamic curves, obtained at 0,20 μA s^{-1} cm^{-2}, for the isopropanol (0.2 mol L^{-1}) oxidation on Pt(111), Pt(100), and Pt(110), in H_2SO_4 (0.10 mol L^{-1}). (B – D). Inset for the potential instabilities

2. Galvanostatic time series of isopropanol electrooxidation reaction on Pt(110) surface

Figure S2 presents the studies of the galvanostatic curve for the isopropanol electrooxidation reaction on Pt(110).

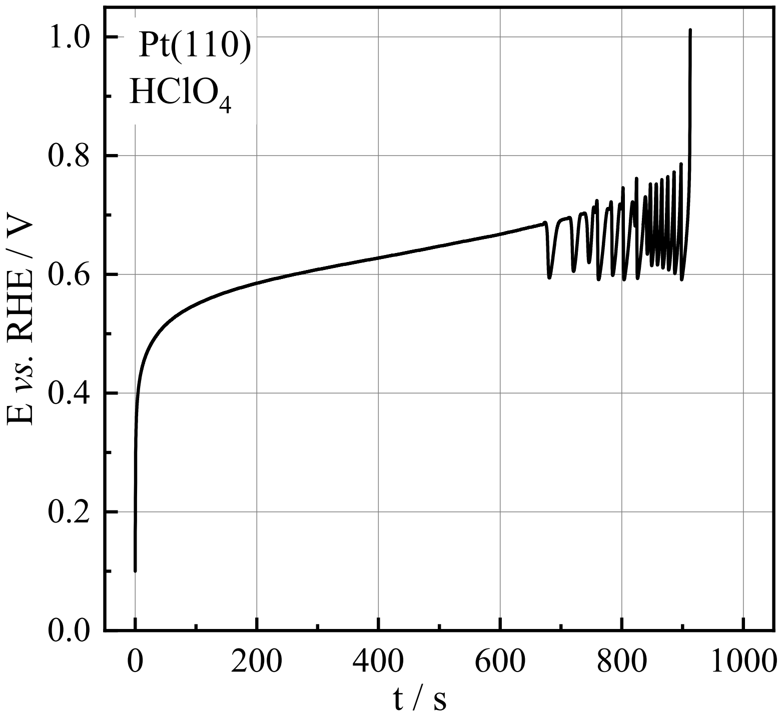

Figure S2. Galvanostatic time series of isopropanol (0.2 mol L^{-1}) electrooxidation reaction on Pt(110) $HClO_4$ (0.1 mol L^{-1}), at 0.18 mA cm^{-2}.

3. Galvanostatic time series of isopropanol electrooxidation reaction on Pt(100) surface

Figure S3 presents the studies of the galvanostatic curve for the isopropanol electrooxidation reaction on Pt(100).

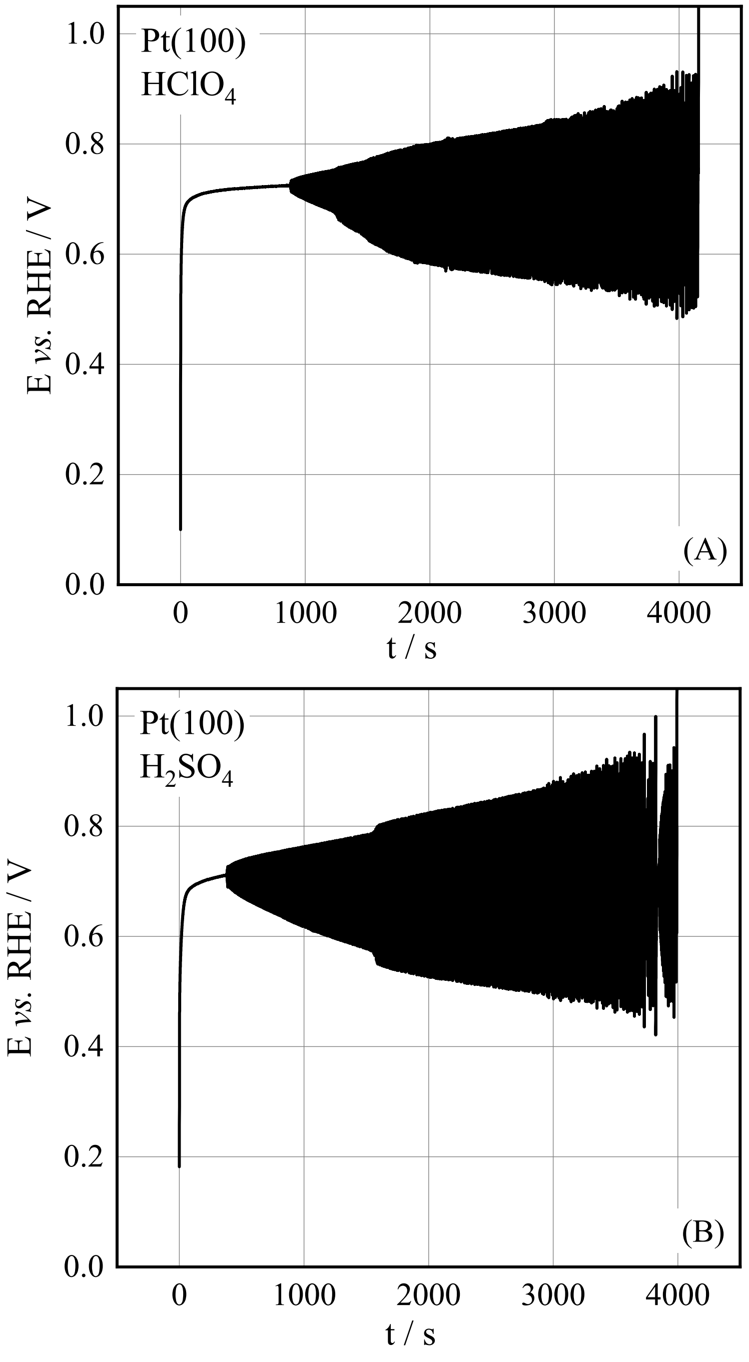

Figure S1. Galvanostatic time series of isopropanol (0.2 mol L^{-1}) electrooxidation reaction on Pt(100) in: (A) $HClO_4$ (0.1 mol L^{-1}) at 0.21 mA cm^{-2} and (B) H_2SO_4 (0.1 mol L^{-1}) at 0.13 mA cm^{-2}.
